# Olfactory impairment and the risk of cognitive decline and dementia in older adults: a meta-analysis

**DOI:** 10.1016/j.bjorl.2020.07.009

**Published:** 2020-09-12

**Authors:** Zirong Chen, Hongbo Xie, Linyin Yao, Yongxiang Wei

**Affiliations:** a Beijing Institute of Heart Lung and Blood Vessel Diseases, Beijing, China; b Capital Medical University, Beijing AnZhen Hospital, Otolaryngology Head and Neck Surgery Department, Beijing, China

**Keywords:** Olfaction disorders, Anosmia, Cognitive declines, Dementia, Meta-analysis

## Abstract

**Introduction:**

The prediction of the impact of olfactory impairment on cognitive decline in older adults has been different among different age groups.

**Objective:**

This meta-analysis sought to estimate the predictive power of olfactory impairment on cognitive decline during follow-up in older adults of different ages.

**Material and methods:**

A medical literature search was carried out using these databases for eligible studies: MEDLINE, COCHRANE and EMBASE. Studies recording olfaction and cognition detection at the beginning and end of the follow-up were included in the preliminary screening. The medical records of older adults without cognitive impairment at the beginning of the follow-up were taken into account in this analysis. Raw data was extracted in order to estimate the relative risk and the corresponding 95% confidence interval (95% CI). Subgroup analysis of age was performed to eliminate the effect of age on the results. Statistical heterogeneity was measured using the *I*^2^ index and Cochran's *Q* test.

**Results:**

Eight studies were enrolled in this analysis (3237 events and 13165 participants), and the pooled relative risk for the 70–80 years old subgroup was 2.00 (95% CI = 1.79–2.23).

**Conclusion:**

Relatively, there is a higher risk of cognitive impairment at the end of follow-up in younger adults with olfactory impairment at the beginning of follow-up. The length of follow-up has a little effect on the relative risk.

## Introduction

Cognitive decline usually appears with aging and has a great impact on daily life, increasing the mortality of the elderly and placing a huge burden on society.^1^, ^2^, ^3^, ^4^ Given the insidious onset of cognitive decline and its slow progressive of transition to dementia, and the fact that there is no effective treatment for this disorder, primary prevention is essential via screening high-risk groups.^5^

Olfactory impairment, especially the impairment of olfactory identification, is considered to be a potential early warning sign of neurodegenerative disorders, such as Parkinson's disease (PD) and Alzheimer's diseases (AD).^6^, ^7^, ^8^, ^9^, ^10^ Anatomical studies suggest that neurofibrillary tangles in the olfactory bulb and in the projection pathways from the olfactory bulb to secondary olfactory brain regions are the earliest pathologic features of AD, which damage olfaction.^6^, ^11^ The deposition of pathogenic proteins, α-synuclein and hyperphosphorylated tau protein, in the olfactory bulb and tract (OBT) weaken synaptic function.^12^, ^13^, ^14^, ^15^, ^16^

OBT atrophy on magnetic resonance imaging (MRI) in AD and mild cognitive impairment is another proof of olfactory impairment.^6^, ^17^ Several studies have confirmed the fact that older adults with olfactory dysfunction present an increased risk of transition to dementia.^18^, ^19^, ^20^, ^21^, ^22^, ^23^

Previous studies have shown that cognitive decline in older adults comes with impaired olfaction or olfactory identification.^24^, ^25^ Cognitive decline becomes evident in the middle-aged (age 45–49),^26^ however, it is only noticed by a few. A systematic review reported the predictive value of olfactory impairment for cognitive decline among cognitively normal adults.^27^ Further analysis of age subgroups was not performed due to limited data. In this review, the author aims to estimate the relative risk (RR) of each age subgroup to explain the predictive power of olfactory impairment for cognitive decline and dementia in different age groups.

## Methods

### Search strategy

The authors carried out this analysis following the criteria of “Meta-analyses of Observational Studies in Epidemiology guidelines”.^28^ An extensive literature search was run to identify studies that recorded olfaction and cognition assessment at the beginning and at the end of follow-up. Participants were residents and the average age in each study was over 50 years old. Participants with cognitive decline were excluded at the beginning of follow-up. Studies that were too short for follow-up were excluded, given the long latency period of the disease. Medline, Cochrane and Embase were used to get identified studies. For the search on outcomes, we identified the articles using medical keywords ([cognitive OR cognition OR dementia OR Alzheimer's disease OR mild cognitive impairment OR Parkinson's disease] AND [olfactory OR olfaction OR odor OR odorant OR smelling OR hyposmia]). The reference lists of retrieved articles were searched for other additional relevant studies.

Data required results enough to construct the 2 × 2 contingency table of diagnostic performance for olfaction test. Olfactory impairment at the beginning of follow-up was defined as the exposure factor. Cognitive decline at the end of follow-up was defined as the positive result. Participants were divided into four groups based on olfaction and cognitive functions and the RR of each group was calculated.

The selected studies provided statistical information that permitted meta-analytical methods to be used. This search procedure yielded eight articles. Details of the participants in each included study are described in the Table 1.

**Table 1:** Baseline characteristics of each study population.

Author year	Participants	Duration (Y)	Samples (outcome)	Sex female (%)	Age (Y)		Education (Y)		OI		N-OI	
					Mean	SD	Mean	SD	Event	Total	Event	Total
Adams DR^18^ 2017	Older adults^a^	5Y	2906 (DE)	51.1	68	7.6	–	–	58	644	66	2262
Devanand DP^30^ 2019	Urban community	4Y	724 (CD)	65.81	75.9	2.7	11.62	2.9	36	165	22	221
				76.47	84.89	3.7	9.67	4.84	58	196	31	142
Fischer ME^31^ 2016	EHLS	10Y	1884 (CD)	59.1	66.7	8.4	–	–	79	323	87	1561
Graves AB^32^ 1999	Community^b^	2Y	1599 (CD)	55.61	71.45	5.34	13	2.88	28	168	116	1 431
Kristine Yaffe^33^ 2017	Community^c^	12Y	1510 (DE, white)	48.34	75.65	2.72	–	–	114	337	164	1173
			918 (DE, black)	58.17	75.42	2.79	–	–	84	263	129	655
Lipnicki DM^34^ 2013	SMAS	1.92Y	572 (CD & DE)	45.9	78.59	4.75	11.68	3.49	37	169	56	496
Rosebud O. R^35^ 2016	Mayo^d^	3.5Y	1430 (CD)	50.6	79.5	5.3	14.3	2.8	120	477	130	953
Stanciu I^36^ 2014	Residents^e^	10Y	1529 (CD)	55.7	61.2	11.7	11	4.1	54	300	105	1229

This table demonstrates the basic data of the participants included in the enrolled studies, consisting of the total number of participants, the length of follow-up, the average age, sex ratio, education years, number of exposed persons (suffering from olfactory impairment), and number of final events (cognitive decline or dementia).

CD, cognitive decline; DE, dementia; OI, olfactory impairment; N-OI, non-olfactory impairment; EHLS, Participants in The Epidemiology of Hearing Loss Study; SMAS, Participants of Sydney Memory and Ageing Study.

a Longitudinal study of a population representative of U.S. older adults.

b A community-based longitudinal study of memory and aging.

c Community-dwelling black and white older adults.

d Participants of Prospective Mayo Clinic Study of Aging.

e A sample of 1529 participants.

### Quality assessment

**Figure 1:**
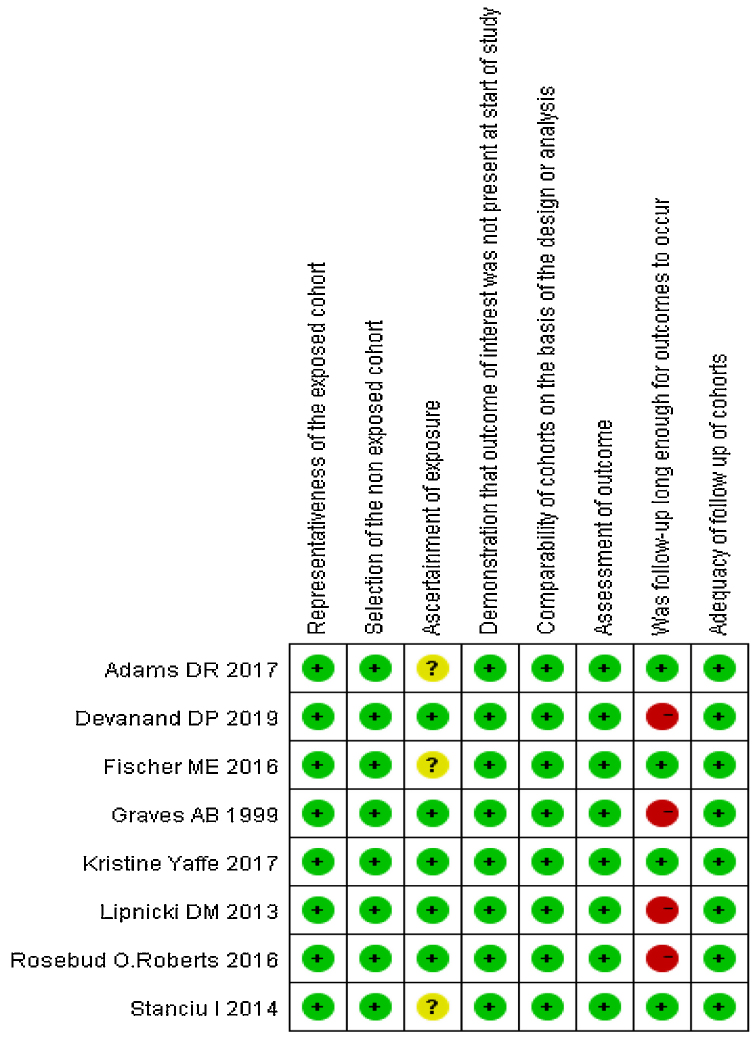
Eight enrolled studies quality evaluation.

The methodological quality of the included studies was assessed by two reviewers independently using the Newcastle-Ottawa Scale (NOS).^29^ Disagreements were resolved in consensus meetings. The details are demonstrated in Fig. 1.

### Data extraction

Two authors independently evaluated the eligibility of all studies retrieved from the databases according to the above selection criteria: the two lists were then compared and disagreements between evaluators were resolved by discussion. We extracted the following data from each publication, using a standardized data form: study name (together with first author's name and the year of publication), study design, study population, country, sample size, study period, results of the studies (case population, adjusted RRs or ORs with their corresponding 95% CIs), instrument of olfactory measurement, method of cognitive assessment, and adjustments for confounding factors in the analysis (Table 1).

### Statistical analysis

Olfactory impairment was considered as the exposure factor while both cognitive decline and dementia were defined as positive results. A 2 × 2 table of diagnostic performance for olfaction testing was constructed to estimate the RR of each study, and to compute a pooled RR with 95% CI.

Statistical heterogeneity was measured using the *I*^2^ index and Cochran's *Q* test. None, low, moderate, and high degrees of heterogeneity were defined as <25%, 25% ≥ 50%, 50% ≥ 75%, and ≥75%, respectively. The null hypothesis that the studies are homogeneous would be rejected if the *p*-value were less than 0.05. The fixed-effect model was used to estimate pooled RR, assuming that the studies included in the meta-analysis had the same effect size. Given the existence of statistically significant heterogeneity, the random-effect model was used to combine the results, assuming that the studies included in the meta-analysis had varying effect sizes across the studies. In this meta-analysis, hazard ratio (HR) and odds ratio (OR) were considered to equivalent to RR as general measures of risk.

Two sensitivity analyses were performed to test the stability of the results: (1) replacing a random effects model with a fixed effects model and (2) removing one primary study from the pooled analysis each time. However, results of the evaluation for asymmetry possess relatively low power to reflect a real publication bias when the total number of studies included in the meta-analysis is small (25 or fewer), which is the case in this review. All analyses were conducted using Review Manager statistical software (version 5.3). A two-sided *p*-value ≤0.05 was considered statistically significant.

## Results

### Study characteristics

**Figure 2:**
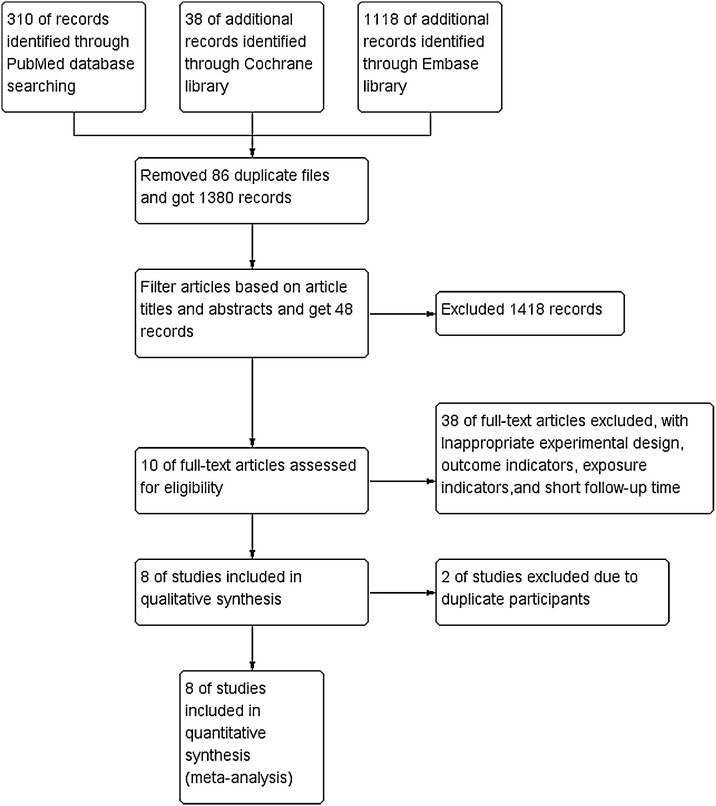
A flow chart showing the process of identifying suitable studies for the meta-analysis.

After reviewing 1466 titles and abstracts, and 43 full articles, 8 articles were enrolled in this meta-analysis.^18^, ^30^, ^31^, ^32^, ^33^, ^34^, ^35^, ^36^ The literature search process can be seen in Fig. 2. Among these, 6 studies were carried out in the United States,^18^, ^30^, ^31^, ^32^, ^33^, ^35^ while the rest of the studies were carried out in Australia and Sweden.^34^, ^36^ The sample size ranged from 572^34^ to 2.906^18^ and the follow-up period ranged from 23 months^34^ to 12 years.^33^ They all included adjusted OR, RR or HR and 95% CI. Details of each study can be seen in Table 1.

Of the 8 studies with data on cognitive decline and dementia, including 13,165 participants and 1574 events, 5 reported results on cognitive decline,^30^, ^31^, ^32^, ^35^, ^36^ 2 on dementia,^18^, ^33^ and 1 reported results on both.^34^

### RR

**Figure 3:**
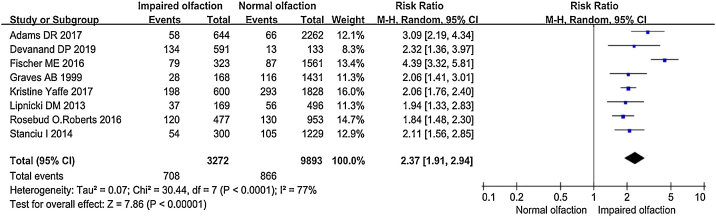
Forest plot of pooled RRs and 95% CIs of cognitive decline in relation to olfactory impairment.

Olfactory impairment was positively associated with a risk of cognitive decline. Comparing normal with impaired olfaction, the risk of cognitive decline was increased by 137% (pooled RR = 2.37; 95% CI = 1.91–2.94) (Fig. 3), which is basically consistent with the review of Windon et al.^27^ However, statistically significant heterogeneity was observed among these studies (*I*^2^ = 77%, *p* < 0.00001). Given that the variability in effect sizes between olfactory impairment populations and normal olfactory populations differed more than would be expected from sampling error alone, analysis of potential moderator variables is necessary.

We obtained pooled estimates by using a random-effects model. Dots indicate the adjusted RRs by comparing impaired with normal olfaction. The size of the shaded square is proportional to the percentage weight of each study. The horizontal lines represent 95% CIs.

### Subgroup analysis

**Figure 4:**
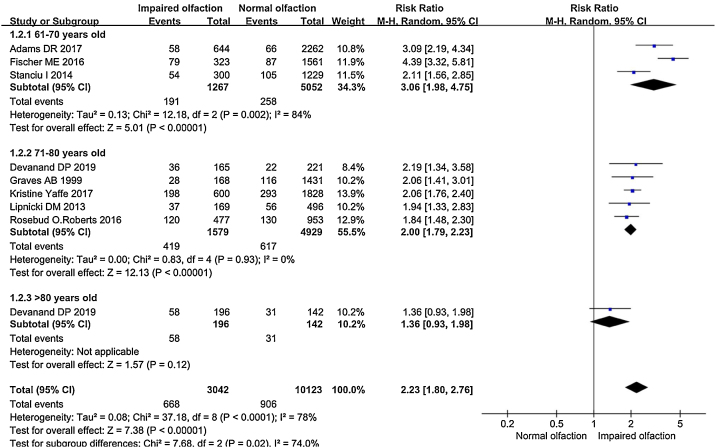
Subgroup analysis forest plot.

In the subgroup analysis by age, the RR of different subgroups were obviously different (Fig. 4). In the 60-years old subgroup,^18^, ^31^, ^36^ the association between olfactory impairment and subsequent cognitive decline was tenacious (RR = 3.06, 95% CI = 1.98–4.75). Due to the limitation of samples, substantial heterogeneity could be seen in the 60-years old group (*I*^2^ = 84%, *p *= 0.002). This association was also significant in the 70-year-old subgroup (RR = 2.00, 95% CI = 1.79–2.23) but weaker than that of the 60-years old subgroup, with no degrees of heterogeneity (*I*^2^ = 0%, *p* = 0.93).^32^, ^33^, ^34^, ^35^, ^37^ The participants of the Devanand^30^ study were divided into different age subgroups. Participants over 80 years old in this study were enrolled in the 80 years old subgroup and the correlation between olfactory impairment and subsequent cognitive decline in this group was slower than that of other groups (RR = 1.36, 95% CI = 0.93–1.98).^30^

All included studies were divided into three subgroups based on the average age of the included population in each study. The RR of the subgroup analysis for the 61–70 years old group was 3.06, the RR for the 71–80 years old group was 2.00, and the RR for the >80 group was 1.36.

### Sensitivity analyses

The fixed effects model was used to test the robustness of analysis outcome. To check the impact of every single study on the pooled RR, we conducted sensitivity analysis by removing one primary study from the analysis every time. Subgroup analyses showed that no single study substantially had an interference on the overall estimates.

## Discussion

This study described the association between olfactory impairment and subsequent cognitive decline and dementia in older adults with normal cognition and suggested that the predictive value is different for different age subgroups. Older adults with olfactory impairment had an almost doubled risk of developing cognitive decline or dementia. These stable associations seemingly persisted across different geographic areas, follow-up periods, sample size, cognitive function assessment methods, and primary study qualities. Previous cross-sectional and longitudinal studies support this opinion and a review confirmed the predictive value of olfactory impairment for cognitive decline or dementia.^35^, ^37^, ^38^ This study showed that younger adults with olfactory impairment were more likely to develop cognitive impairment or dementia by the end of follow-up.

The strengths of this analysis are as follows: all the enrolled studies were longitudinal cohort studies, which have a more credible value, and the follow-up period was over 20 months, which eliminates recall bias.

There were some limitations that existed in this analysis which created the statistical heterogeneity. Different olfactory detection methods were used in the enrolled studies. The Brief Smell Identification Test (B-SIT),^39^ also called the Cultural-Cross Smell Identification Test (CC-SIT), was widely used worldwide. The Validated 5-item test, Subjective olfactory complaint and San Diego Odor Identification Test (SDOIT)^40^ were also used in the enrolled studies.^31^ These detection methods have been proven effective. Some of these tests are adapted from the Pennsylvania Smell Identification Test (UPSIT).^41^ There are subtle differences in the cutoffs, which contributed to the statistical heterogeneity. Strict and accurate standards were used in each study to assess cognitive functions. The Mini Mental Status Exam was the commonly used among the enrolled studies. All the assessment methods in each study are demonstrated on the table of characteristics of studies (Table 1).

The subgroup with a younger average age showed a higher RR factor of olfactory impairment on cognitive decline or transition to dementia. Participants with olfactory impairment in the 60–70 years old subgroup had 2.64–5.27 times the probability of cognitive decline or transition to dementia compared to those with normal olfaction. In a 10 year follow-up study, cognitive decline occurred in the middle-aged (baseline age 45–50 years), and the group with a baseline age of 64–70 years experienced a greater decline in cognitive function after 10 years, with a 9.6% decrease compared to baseline.^26^ The estimated prevalence of mild cognitive impairment in population-based studies ranges from 10% to 20% in persons older than 65 years of age.^42^, ^43^ Age may contribute mostly to the highest RR of this subgroup in comparison to other subgroups. The limited sample size equally contributed to the heterogeneity.

The follow-up duration for the 70–80 years old subgroup varied between 23 months and 12 years, with a stable RR from 1.84 to 2.11. This showed that there was no linear correlation between follow-up time and the RR of olfactory impairment on cognitive function. Particularly, the RR did not increase significantly with the extension of follow-up time, which is inconsistent with the results of previous systematic reviews.^27^ In studies with a follow-up duration of more than ten years,^33^, ^36^ both the RR calculated from the original data and the adjusted RR were not significantly different from those of studies with short follow-up duration.^32^, ^34^

Previous studies have suggested that olfactory impairment is associated with cognitive decline, and the impairment of OBT. The projection pathways from the olfactory bulb to the secondary olfactory brain regions were equally highly correlated with neurofibrillary tangles and pathogenic proteins such as α-synuclein and hyperphosphorylated tau protein in these areas, which damage olfaction and contribute to the early pathogenic process of neurodegenerative diseases such as AD and PD. Consequently, olfaction is regarded as an effective and valuable tool for early detection of neurodegenerative diseases, and it can be an indicator for future cognitive function.

Due to the limitation of detection methods for dementia and cognitive decline in the designs of the enrolled studies, it is inconvenient in distinguishing persons with a high risk of cognitive impairment from those not at risk. Nevertheless, the pathological changes in OBT and other olfaction related areas are warning signs for cognitive decline. Olfaction is an outcome of the pathological changes in these areas, which may contribute to the prevention of neurodegenerative diseases. Olfactory training gives a positive future for improvement of cognition. Early screening and preventive treatment may slow disease progression (Table 2).

**Table 2:** Detection methods.

Author year	OT*-cutoff	CogT	OI		Multivariate logistic regression
			Adjusted RR	95% CI	
Adams DR^18^ 2017	Validated 5-item test	Physical exam^a^	2.13	1.32–3.43	Age, gender, race/ethnicity, education, comorbidities, initial cognition
Devanand P^30^ 2019	B-SIT – 9/12	NP-test^b^	2.48	1.34–4.58	Age, gender, education, language
					
Fischer ME^31^ 2016	SDOIT – 6/8	MMSE	4.18	2.68–6.51	Age, sex, education
Graves AB^32^ 1999	CC-SIT – 6/12	CASI	2.92	1.76–4.86	No-adjustment
Kristine Yaffe^33^ 2017	B-SIT – 9/12	Modified MMSE	3.34	2.45–4.54	Age, education, APOE ɛ4 allele, depression, smoking, physical activities, BMI
			2.03	1.44–2.84	
Lipnicki DM^34^ 2013	B-SIT – 9/12	International criteria^c^	1.86	1.15–3.01	Sex, marital status, BSIT score, and age
Rosebud O. R^35^ 2016	B-SIT – 9/12	Physician^d^	1.85	1.43–2.39	Sex and education, with age as the time scale
Stanciu I^36^ 2014	Subjective olfaction	MMSE	2.17	1.40–3.37	Age, gender, years of education, MMSE, olfaction, and subjective olfaction

This table shows the detection methods used in each study to detect the olfaction and cognitive function. The value of the cutoff of different olfactory detection methods are shown in the table. And it also shows the adjusted relative risk coefficient and 95% confidence interval in each study, as well as the variables included in the multiple regression analysis.

OT, olfaction test; CogT, cognition test; B-SIT, Brief Smell Identification Test (B-SIT)^36^; SDOIT, San Diego Odor Identification Test (SDOIT)^37^; CC-SIT, Cultural-Cross Smell Identification Test; UPSIT, University of Pennsylvania Smell Identification Test^38^; BMI, Body Mass Index; MMSE, Mini-mental State Examination; CASI, Cognitive abilities screening instrument.

a Low-cost component of the physical examination, proxy interviews.

b A standardized neuropsychological test battery.

c A panel of psychogeriatricians, neuropsychiatrists and clinical and research neuropsychologists using current international consensus criteria.

d Evaluated by a physician.

## Conclusion

This study gathered evidence supporting the fact that impaired olfactory function is significantly associated with the risks of cognitive decline and dementia in older adults. It is necessary to give more attention to persons aged 60–70 years with olfactory disorders because of the higher associated risk factor. Follow-up duration does not have a significant effect on RRs. Considering its advantages of safety, cost-effectiveness, and ease to test and interpret, olfactory tests may be effective indicators of the development of cognitive decline and dementia in older adults.

### Limitations

More large-scale cohort studies are needed to determine age, gender, and cutoffs of olfactory testing to enhance the effectiveness and efficiency of an early indicator of cognitive impairment and dementia, in the future. Olfactory impairment is moderately associated with cognitive decline, and more auxiliary detection methods are needed to improve prediction for cognitive decline. The analysis of pooled RR was limited since it was calculated from original data without adjustment.

## Funding

This work was supported by the Beijing Municipal Hospital Scientific Research Training Program (PX2019023).

## Conflicts of interest

The authors declare no conflicts of interest.
